# Various Silver Nanostructures on Sapphire Using Plasmon Self-Assembly and Dewetting of Thin Films

**DOI:** 10.1007/s40820-016-0120-6

**Published:** 2016-11-28

**Authors:** Sundar Kunwar, Mao Sui, Quanzhen Zhang, Puran Pandey, Ming-Yu Li, Jihoon Lee

**Affiliations:** 1 College of Electronics and Information, Kwangwoon University, Nowon-gu, Seoul, 01897 South Korea; 2 Institute of Nanoscale Science and Engineering, University of Arkansas, Fayetteville, AR 72701 USA

**Keywords:** Ag nanostructures, Surface plasmon, Self-assembly, Dewetting

## Abstract

Silver (Ag) nanostructures demonstrate outstanding optical, electrical, magnetic, and catalytic properties and are utilized in photonic, energy, sensors, and biomedical devices. The target application and the performance can be inherently tuned by control of configuration, shape, and size of Ag nanostructures. In this work, we demonstrate the systematical fabrication of various configurations of Ag nanostructures on sapphire (0001) by controlling the Ag deposition thickness at different annealing environments in a plasma ion coater. In particular, the evolution of Ag particles (between 2 and 20 nm), irregular nanoclusters (between 30 and 60 nm), and nanocluster networks (between 80 and 200 nm) are found be depended on the thickness of Ag thin film. The results were systematically analyzed and explained based on the solid-state dewetting, surface diffusion, Volmer–Weber growth model, coalescence, and surface energy minimization mechanism. The growth behavior of Ag nanostructures is remarkably differentiated at higher annealing temperature (750 °C) due to the sublimation and temperature-dependent characteristic of dewetting process. In addition, Raman and reflectance spectra analyses reveal that optical properties of Ag nanostructures depend on their morphology.

**Electronic supplementary material:**

The online version of this article (doi:10.1007/s40820-016-0120-6) contains supplementary material, which is available to authorized users.

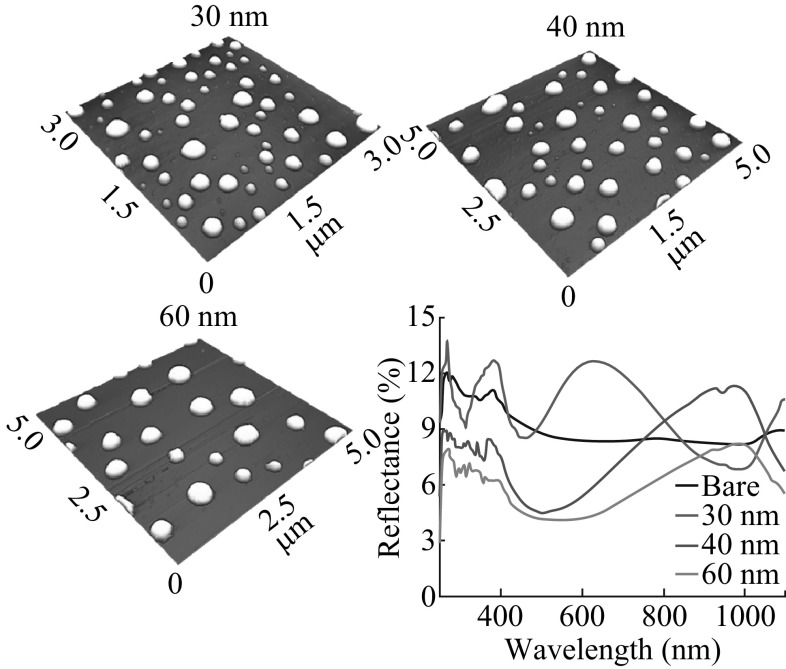

## Highlights

- Various configurations of Ag nanostructures were systematically fabricated on sapphire (0001) by controlling the deposition thickness and annealing environment in a plasma ion coater.
- The results were systematically analyzed based on the solid-state dewetting, surface diffusion, Volmer-Weber growth model, coalescence and surface energy minimization mechanism.

## Introduction

Silver (Ag) nanostructures, the dimension range within nanoscale with definite geometric shape, size, and configuration such as nanoparticles (NPs), nanoclusters, and nanowires, have been widely used in optical, electronic, catalytic, sensing, and biomedical devices [1–6]. Such devices utilize Ag nanostructures for the improved performances, i.e., enhanced scattering and absorption of light, high electrical conductivity, and enhanced sensitivity due to the strong surface plasmon resonance exhibited by the Ag nanostructures [7–9]. Furthermore, the large surface-to-volume ratio, selective binding, and detection with specific target enable Ag nanostructures to be applicable as the catalysis as well as the chemical and biological sensors [5, 10–13]. In specific, the increased photocurrent density due to the high electromagnetic field strength in the vicinity of excited surface plasmons can enhance the power conversion efficiency of organic solar cells [14]. The electro spun polymer nanofibers immobilized with Ag NPs can exhibit the superior catalytic reduction with high efficiency and reusability [15]. Generally, metallic nanostructures show the controllable configuration, shape, size, and distribution. For instance, photoelectron lifetime of TiO_2_ nanotube arrays decorated by Ag NPs can be enhanced because of size-dependent localized surface plasmon resonance, which in turn can maximize the photo conversion efficiency [16]. Although several methods for the Ag nanostructures synthesis have been practiced, thermal approach taking the advantage of solid-state dewetting of the thin film is still a relatively simple and cost-effective approach to control the configuration, shape, and size of the Ag nanostructures [17–21].

Sapphire has been successfully used in optical devices such as light-emitting diode, laser diodes, and IR-UV detector owing to its wide transparency window from 180 to 5500 nm, wide band-gap, and thermal, chemical, mechanical stability. Therefore, the systematic characterization of Ag nanostructures on sapphire (0001) may be very important for novel applications, which is rarely reported up to date. In this work, we demonstrate the configurational and dimensional transformation of Ag nanostructures on sapphire (0001) via the systematic control of deposition thickness in various annealing environments. The evolution begins with the self-assembly of diffused Ag adatoms at sufficient thermal energy. With the controlled deposition amount, the tiny to the enlarged dome-shaped NPs, the merged nanoclusters, Ag nanocluster networks were fabricated. On the other hand, for the identical deposition range of Ag thin films annealed at a higher temperature, Ag nanostructures show distinct evolution such as the formation of tiny round NPs, round and widely spaced large Ag NPs, and elongated Ag NPs due to the substantial sublimation and high diffusion rate. Furthermore, the optical characteristics of Ag nanostructures were studied by the Raman and reflectance spectra.

## Experimental Section

### Substrate Preparation

The systematic study of Ag nanostructures was performed on 430-micron-thick *c*-plane sapphire (0001) with off-axis ±0.1° (iNExus Inc, South Korea). Prior to the fabrication, the wafers were diced into small uniform pieces using mechanical saw and subjected to the degassing in a pulsed laser deposition (PLD) chamber to remove the gaseous and particle contaminants. The degassing was performed at 600 °C for 1800 s under 1 × 10^−4^ Torr. Figure S1 presents the surface morphology of sapphire after the degassing with smooth surface morphology and the Raman spectra depicts the five active phonon modes of the sapphire.

### Fabrication of Ag Nanostructures

In order to investigate the evolution of Ag nanostructures, the deposition amount of Ag was systematically varied at a fixed and distinct annealing condition. Various amounts of Ag were deposited on substrates by sputtering in a plasma ion coater. The thickness of Ag films was controlled by the deposition time at a growth rate of 0.1 nm s^−1^ with ionization current of 5 mA under 1 × 10^−1^ Torr. After the deposition, the uniform distribution of Ag atoms on substrates was confirmed by the atomic force microscope (AFM) scanning before annealing. The surface morphology became rougher with higher deposition thickness as shown in Fig. S2. Two series of samples with identical thickness between 2 and 200 nm were prepared. Then, the as-deposited samples were subsequently annealed at distinct temperatures of 550 and 750 °C with the linear ramping rate of 4 °C s^−1^ in the PLD chamber under 1 × 10^−4^ Torr for 180 s. Then the system temperature was quenched down to the ambient.

### Characterization

The morphologies of the as-prepared Ag nanostructures were carried out by AFM (XE-70, Park Systems Corp., South Korea) in an ambient condition with a non-contact mode. For the consistency of measurement and minimal tip effect, all the samples were scanned using the NSC16/AIBS tips with a drive frequency of ~270 kHz. The surface morphologies and the evolution of Ag nanostructures were analyzed by XEI software in terms of top-views, side-views, cross-sectional line profiles, Fourier filter transform (FFT) spectra, surface area ratio (SAR), and RMS roughness (*R*_q_). Larger-scale surface morphology was investigated by a scanning electron microscope (SEM, CX-200, COXEM, South Korea). The elemental characterization of samples was performed by an energy-dispersive X-ray spectroscope (EDS, Noran System 7, Thermo Fisher Scientific, USA). The Raman spectra were measured using UNIRAM II (UniNanoTech Co. Ltd, South Korea) with a CW 532-nm laser at 220 mW excitation. Reflectance spectra were obtained using deuterium light for UV region and halogen light for visible and NIR region. The optical measurements were carried out at in an ambient condition in dark room.

## Results and Discussions

**Fig. 1:**
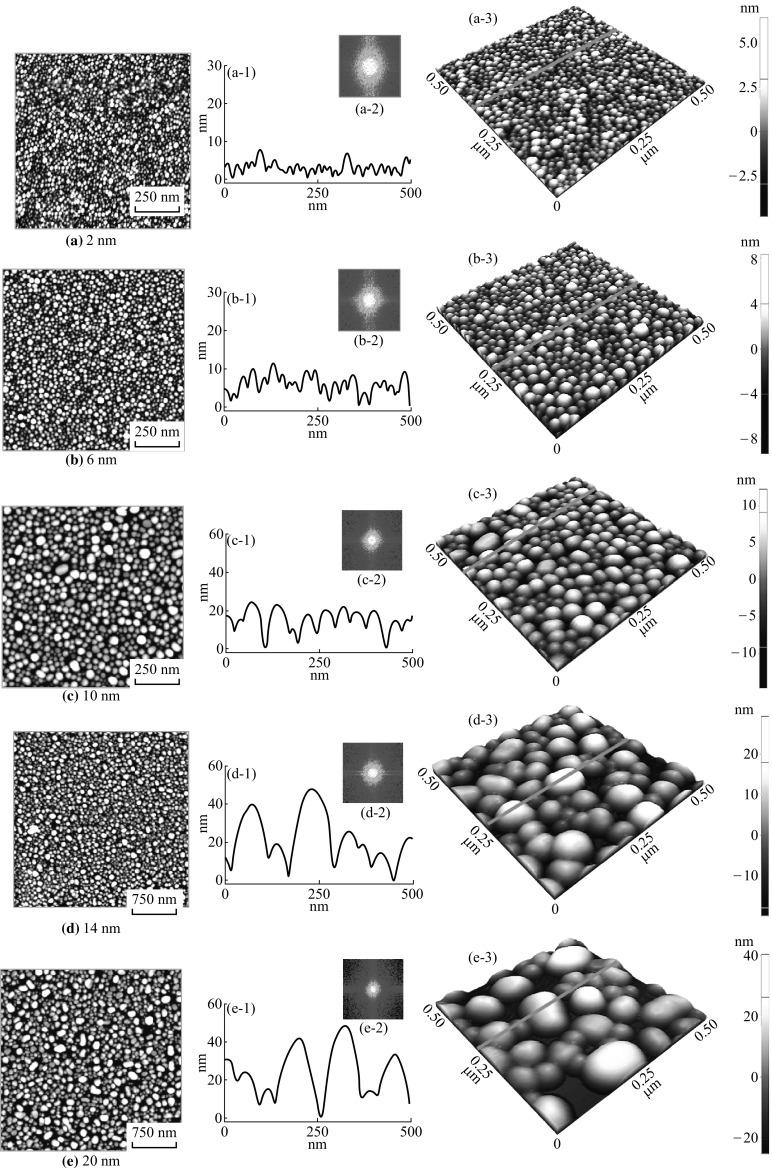
As-prepared Ag nanoparticles (NPs) on sapphire (0001) based on the Ag thickness variation between 2 and 20 nm annealed at 550 °C for 180 s. AFM top-views of 1 × 1 µm^2^ in **a–c** and 3 × 3 µm^2^ in **d**–**e**. **a-1** to **e-1** Cross-sectional line profiles in reference to the side-views (500 × 500 nm^2^) in **a-3** to **e-3**. **a-2** to **e-2** FFT spectra of the images in **a**–**e**

Figure 1 presents the systematic evolution of the tiny to the enlarged semi-spherical Ag NPs based on the control of Ag film thickness between 2 and 20 nm. The annealing was performed at 550 °C for 180 s. Generally, as depicted from AFM top-views, the Ag NPs are gradually evolved from tiny compact at 2 nm to enlarged and mildly spaced at 20 nm of initial film thickness. In terms of the shape, the Ag NPs possess round dome shape for film thickness from 2 to 20 nm. Initially, the room temperature sputter-grown thin Ag film consists of vacancies, and grain boundaries as well as sapphire (0001) substrate can contain defects, steps, and edges where dewetting can be preferentially initiated upon thermal annealing [22]. Here, the solid-state dewetting of as-deposited thin film can occur by the diffusion of thermally energized Ag adatoms below the melting point. The Ag adatoms undergo diffusion due to the Brownian motion as a consequence of thermal annealing [23]. Consequently, the total surface energy and interfacial energy minimization associated to the Ag films and sapphire (0001) drive the dewetting phenomena [24]. At the annealing temperature of 550 °C, the Ag adatoms can be sufficiently diffused and nucleated to form the Ag nanoparticles with the reduced surface energy. The temperature-dependent surface diffusion *D*_s_ of Ag adatoms can be expressed as Eq. 1,

1 $$D_{s} \propto { \exp }\left( {{{ - E_{\text{Ag}} } \mathord{\left/ {\vphantom {{ - E_{\text{Ag}} } {kT}}} \right. \kern-0pt} {kT}}} \right)$$

where *E*_Ag_ is the activation energy; *k* is the Boltzmann constant; and *T* is the annealing temperature. For the constant annealing temperature, the diffusion length *l*_D_ associated to the Ag adatoms can be constant as explained by Eq. 2,

2 $$l_{\text{D}} = \sqrt {D_{\text{S}} t}$$

where *t* is residence time [25, 26].

From the above two relations, the diffusivity and the diffusion length *l*_D_ can be constant for all the samples due to the constant annealing environment. Initially, at the deposition of 2 nm, the tiny and highly compact 3D Ag NPs were fabricated due to the stronger interatomic interaction between Ag adatoms than that between the adatoms and the sapphire atoms as shown in Fig. 1a. As a result, once the nuclei form at low energy sites, they absorb the diffusing Ag adatoms due to strong bonding energy [27]. Therefore, the evolution of 3D Ag NPs can be explained on the basis of the Volmer–Weber growth model [28]. For the 3D island growth, the surface and interface energy enforces the dewetting by the condition: *γ*_sapphire_ < *γ*_Ag_ + *γ*_interface_, where *γ*_sapphire_ is the surface energy of substrate; *γ*_Ag_ is the surface energy of the film; and *γ*_interface_ is the interfacial energy. Similarly, the short-range intermolecular forces (van der Waals forces) correspondingly govern the self-assembly of the Ag NPs which in turn enhance the dewetting process [29]. Consequently, the equilibrium morphology with reduced free energy can be obtained by the Ag NPs, and the overall energy of the thermodynamic system can be minimized [30].

After increasing the film thickness, the surface morphology developed into the enlarged semi-spherical (dome) NPs, and eventually increased the spacing between the neighboring NPs. As the thickness of Ag film increases, coalescence between adjacent Ag NPs occurs, resulting in the formation of enlarged NPs and the decreasing in NP density [31]. During the coalescence, relatively smaller NPs are ripened, in other words, small NPs can be absorbed by the large ones due to the difference in the surface energy. As a result, NPs’ size keeps increasing with the increased initial film thickness that was driven by the total surface energy minimization mechanism [32]. On the other hand, the weak intermolecular forces like Van der Waal forces between Ag NPs lead the self-assembly process more pronounced in order to attain the equilibrium with lowest energy configurations. Meanwhile, the Ag NPs exhibit dome-shaped structures with an isotropic energy distribution, which can be the natural adaption of total surface energy minimization by the nanostructures [33, 34].

**Fig. 2:**
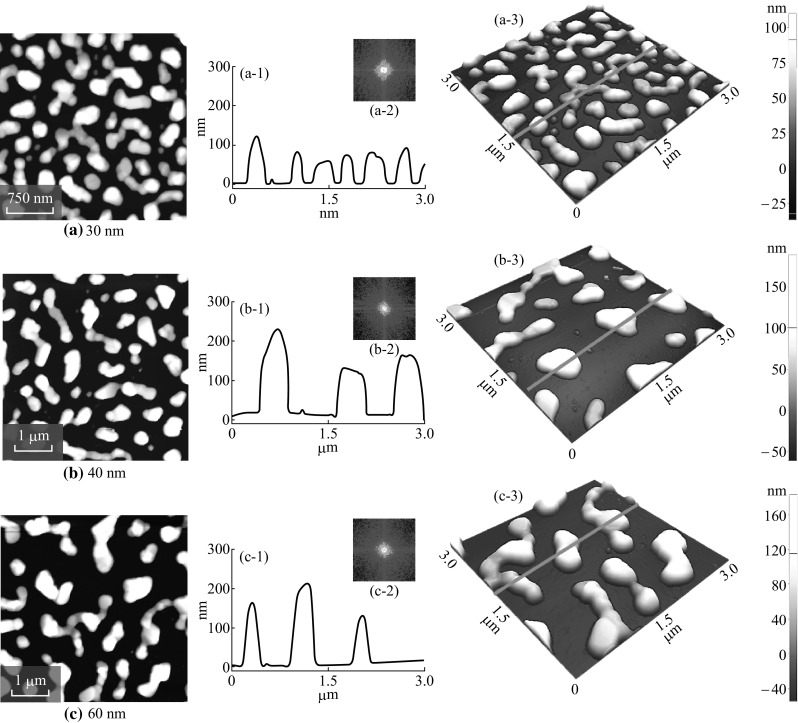
Evolution of merged and irregular Ag nanoclusters on sapphire (0001) with various thicknesses (30–60 nm) of Ag annealed at 550 °C for 180 s. AFM top-views of 3 × 3 µm^2^ in **a** and 5 × 5 µm^2^ in **b**–**c**. **a-2** to **c-2** Cross-sectional line profiles with respect to the line in **a-3** to **c-3**. **a-2** to **c-2** FFT power spectra. **a-3** to **c-3** AFM side-views of 3 × 3 µm^2^

**Fig. 3:**
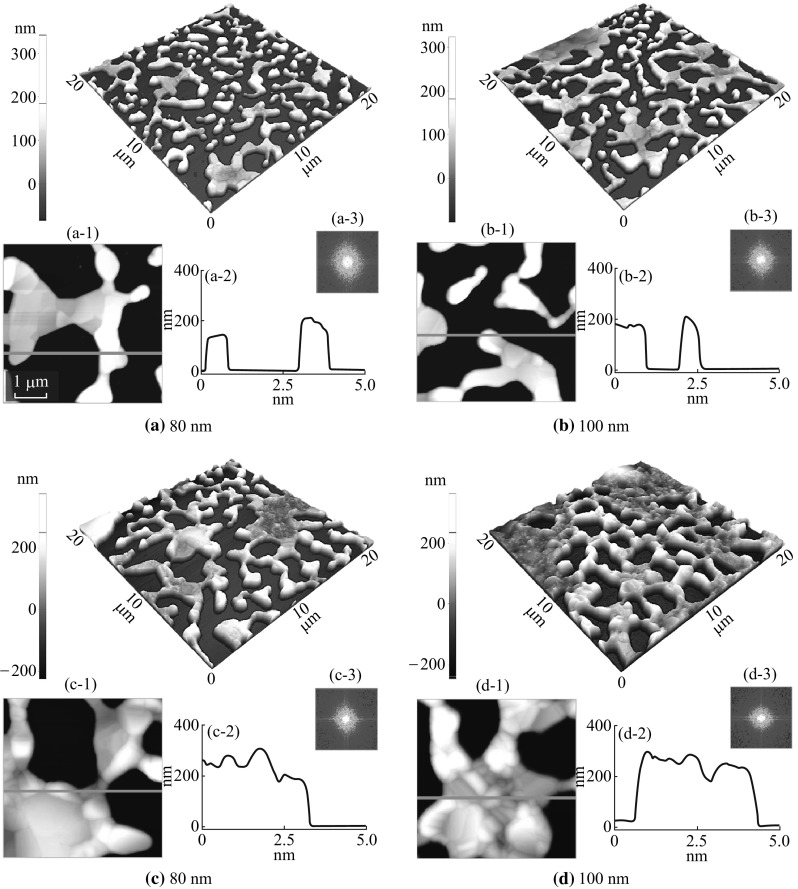
Formation of Ag nanoclusters network on sapphire (0001) with various thicknesses (80–200 nm) of Ag annealed at 550 °C for 180 s. **a**–**d** AFM 3-D side-views of 20 × 20 µm^2^. **a-1** to **d-1** AFM top-views of 5 × 5 µm^2^. **a-2** to **d-2** Cross-sectional line profiles of corresponding AFM top-views. **a-3** to **d-3** 2D FFT power spectra

The consequence of Ag NP evolution is more clearly depicted in the small-scale AFM side-views in Fig. 1a-3 to e-3. The vertical bars of 3D side-views represent the increased range of height with the color variation. For instance, with the deposition thickness of 2 nm, the color variation ranges from −2.5 to 5 nm, whereas for 20 nm the range extended from −20 to 40 nm. Furthermore, the overall morphological enhancement can be correspondingly observed in terms of the cross-sectional line profiles and 2D FFT power spectra as shown in Fig. 1a-1 to e-1 and a-2 to e-2. The cross-sectional line profiles represent the height profiles of surface in reference to the line drawn in corresponding AFM side-views. As suggested by the line profiles, the average height of the NPs is gradually elevated such as ~6 nm for 2–40 nm for 20 nm. Similarly, the dome-shaped cap of the line profiles is gradually expanded horizontally and vertically as the initial film thickness is increased, which shows the simultaneous growth of Ag NPs in both directions. The 2D FFT power spectra, that denote the height distribution of overall surface, uniformly reduce in size with the higher degree of film thickness.

**Fig. 4:**
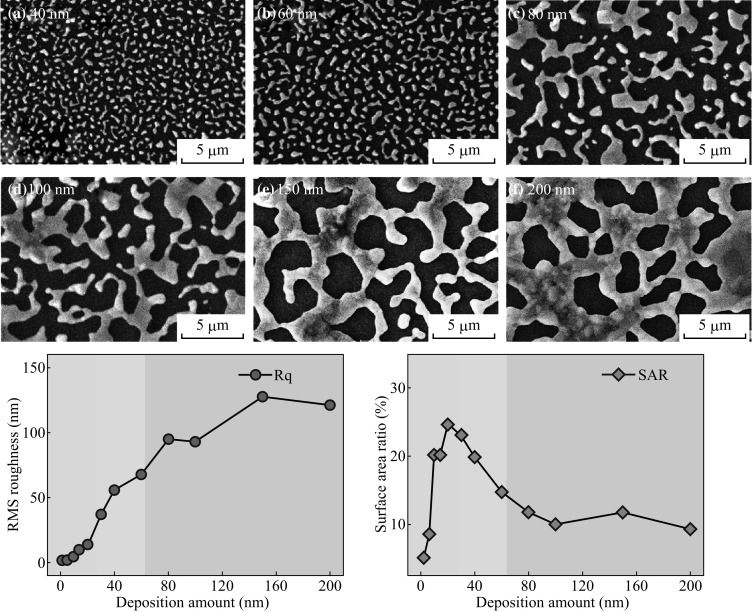
**a**–**f** SEM images of Ag nanostructures fabricated at 550 °C for 180 s. **g** RMS roughness (*R*_q_) with respect to the deposition amount. **h** Surface area ratio (SAR)

As the overall height distribution reduces due to the fabrication of large semi- spherical NPs, the FFT spectra also show the round pattern and contract in size. In addition, the surface morphologies were investigated and quantified by the surface parameters in terms of RMS roughness (*R*_q_) and surface area ratio (SAR). Here, the *R*_q_ provides the average of surface profile height (*y*_*i*_) as Eq. 3,

3 $$R_{\text{q}} = \sqrt {\frac{1}{n}\mathop \sum \limits_{i = 1}^{n} y_{i}^{2} }$$

and the SAR provides the increment of surface area (*A*_s_) with respect to the geometric area (*A*_g_) i.e., x–y plane as SAR = (*A*_g_ − *A*_s_)/*A*_g_. As shown in the *R*_q_ and SAR plots in Fig. 4g, f and summarized in Table S1, the magnitude of both parameters gradually was elevated with respect to the deposition thickness due to the surface enhancement by the formation of much enlarged Ag NPs.

Figure 2 shows the formation of merged Ag nanostructures due to the added initial film thickness between 30 and 60 nm at 550 °C for 180 s. With further increment of deposition thickness, merged and irregular Ag nanostructures were fabricated as shown in Fig. 2a–c. As already discussed, for the higher concentration of Ag adatoms the coalescence phenomena can be further enhanced, and the dewetting of film can be limited due to the film thickness [35]. The dewetting of the process can be influenced by the several parameters, such as initial film thickness, atomic concentration, temperature, and substrate interaction [36]. In terms of the void growth rate (*V*_void_), the annealing temperature and the initial film thickness can be correlated to Eq. 4,

4 $$V_{\text{void}}\,\,\alpha\; \frac{{D_{\text{s}} }}{T} \frac{1}{{t_{\text{f}}^{3} }}$$

where *D*_s_ is the diffusion coefficient; *T* is the temperature; and *t*_f_ is the film thickness [37]. From Eq. 4, the inverse relationship of the void growth and film thickness can be established. Therefore, with increased initial film thickness the void growth can be reduced and vice versa, and as a result overall dewetting process can be slowed down. For the dewetting of thin film, the critical void radius should be less than the void radius as *r*_void_ > *r*_crit_. However, with large film thickness the interfacial energy can be increased such that the wettability can be promoted instead of dewetting due to the increased critical void radius, i.e., *r*_void_ < *r*_crit_. At the same time, the atomic concentration of Ag increases with higher film thickness, and NPs size gets larger by absorbing the nearby tiny particles, and the mobility of the NPs can be reduced [30]. As a result, the Ag NPs can be merged with adjacent ones, and the elongated Ag nanoclusters can be evolved in order to minimize the surface-free energy. For the 30 nm sample, the NPs are merged together exposing more area of the substrate, and the irregular nanostructures are formed. Consequently, with further increased initial film thickness, more elongated irregular nanostructures are evolved and the density is significantly reduced. At the same time, the nanostructures are vertically grown with increased height as shown in Fig. 2a-3 to c-3. The overall surface morphologies are shown by the large-scale SEM images in Fig. 4a, b. Similarly, the average height of cross-sectional line profiles increased with the samples at higher initial film thickness as shown in Fig. 2a-1 to a-2. The FFT power spectra slightly reduce when the deposition varies from 30 to 60 nm as a result improved height distribution with the formation of merged nanostructures. Although the Ag nanostructures are elongated and irregular, the overall height distribution is further reduced as the nanostructures merged together at higher initial film thickness. The corresponding increment in the *R*_q_ from 36.88 to 67.86 nm when deposition varied from 30 to 60 nm explains the surface enhancement with increased height of the Ag nanoclusters as shown in Fig. 4d. However, in case of SAR, it gradually declines from 23.1% at 30 nm to 14.7% at 60 nm as the density of Ag nanostructures is continuously decreased.

Figure 3 depicts the evolution of Ag nanoclusters network with the higher thickness of Ag from 80 to 200 nm after annealing at 550 °C for 180 s. Due to the thicker layer of Ag at the constant temperature and duration, the dewetting process can be further limited along with the enhanced coalescence of nanoclusters and as a result, the Ag nanoclusters developed into polycrystalline finger-like nanostructures [35]. Likewise, the absorption of nearby adatoms and relatively small clusters leads to the merging of nanoclusters and finally the cluster networks can be formed. As shown in Fig. 3a, the Ag nanoclusters start to connect each other. With the further addition of deposition thickness up to 200 nm, smaller nanoclusters are attached in the large ones and the nanocluster fingers are grown, resulting in the network-like structures as shown in Fig. 3b–d.

The large-scale SEM images in Fig. 4c–f clearly present the nanocluster network evolution. Similarly large-scale top-views are shown in Fig. S3. The cross-sectional line profiles depict the average surface heights, which is slightly increased for the higher deposition samples as shown in Fig. 3a-1 to d-1. Furthermore, the FFT power spectra show almost similar pattern and size for the samples with deposition thickness from 80 to 200 nm as a result of similar surface height distribution. The surface modulation by the formation of nanoclusters network can be correspondingly explained in terms of *R*_q_ and SAR. The *R*_q_ value is gradually increased for the higher deposition samples that denote the corresponding rise in nanoclusters height as shown in Fig. 4e. But the SAR value remains almost similar between 80 and 200 nm, which indicates that the surface area of nanocluster networks is not significantly enhanced with respect to the geometric area.

**Fig. 5:**
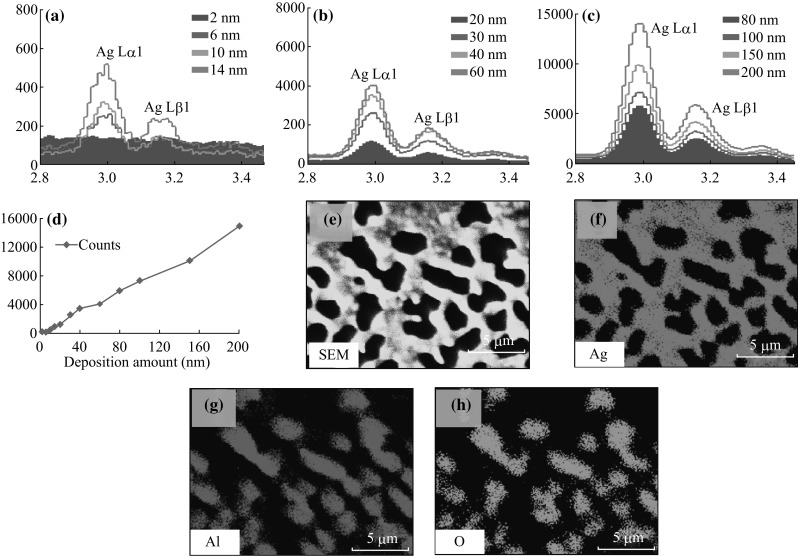
EDS spectra and phase mapping. **a**–**d** Evolution of Ag Lα1 and Ag Lβ1 peaks with respect to the deposition amount varying from 2 to 200 nm. **e** SEM image, **f** Ag phase, **g** Al phase, and **h** O phase of the 200 nm sample annealed at 550 °C for 180 s

Figure 5 presents the elemental characterization of samples with various morphologies. In general, the peaks correspond to Ag, i.e., Ag Lα1 (2.984 keV) and Ag Lβ1 (3.15 keV) are consistently increased with the rise in initial film thickness as shown in Fig. 5a–c. The appropriate ratio of Ag in samples can be confirmed by the linearly increased Ag Lα1 counts with respect to the film thickness as represented by the plot in Fig. 4d. From the quantity analysis, the peaks count of Ag Lα1 for the 2 nm samples is ~186 and it increases to ~14,949 for 200 nm samples. Furthermore, full range EDS spectra depict the oxygen, aluminum, and silver peaks as shown in Fig. S5. In addition, EDS phase maps for the typical 200 nm sample are presented in Fig. 5e–h. In particular, Fig. 5a represents the SEM image and Fig. 5f–h shows the Ag (pink), Al (red), O (green) phase of the corresponding SEM images. The phase map clearly shows the distribution of elements in the sample as shown in Fig. 5e–h. The morphology and Ag phase exactly matches with each other where the dark region denotes the absence of Ag, i.e., the void region in the nanocluster network. For the Al and O phase maps, the bright shapes which are shown in Fig. 5g–h resemble the void region in SEM images. In addition, phase mapping for relatively lower deposition samples is presented in Fig. S4.

**Fig. 6:**
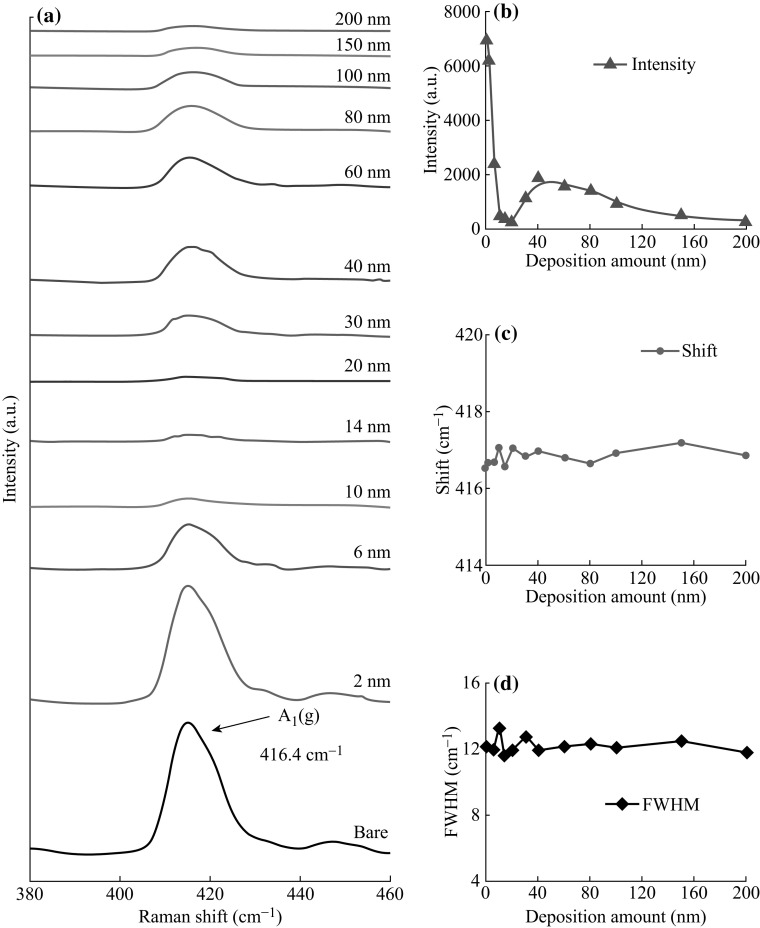
Raman spectra of samples with the various thicknesses (2-200 nm) of Ag annealed at 550 °C for 180 s. **a** Most intense *A*_1_(g) vibration mode at 416.4 cm^−1^. **b**–**d** Peak intensity, peaks shift, and full width at half maximum (FWHM) of the *A*_1_(g)

**Fig. 7:**
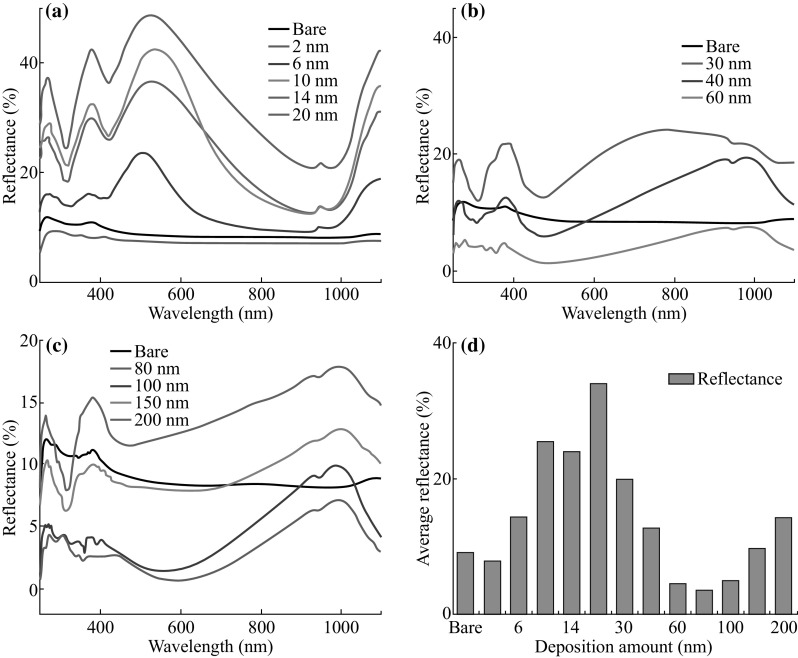
Reflectance spectra of the samples with the various thicknesses (2–200 nm) of Ag annealed at 550 °C for 180 s. **a** 2–20 nm, **b** 30–60 nm, **c** 80–200 nm, and **d** average reflectance summary with respect to the deposition amount

Figures 6 and 7 show the Raman and reflectance spectra of Ag nanostructures by the control of Ag thickness between 2 and 200 nm at 550 °C. In specific, Fig. 6 summarizes the optical characterization based on the Raman spectra, and the peak intensity, peak shift, and full width at half maximum (FWHM) with respect to the film thickness. In general, five Raman bands for bare sapphire (0001) were observed by the excitation of 532 nm laser with the power level of 100 mW. In which, peak at 416.53 cm^−1^ is due to the *A*_1g_ and peaks at 378.24, 446.83, 575.78, and 749.65 cm^−1^ are due to the *E*_g_ vibration modes of the sapphire (0001) [38, 39]. The Raman characteristics of each sample are systematically analyzed on the basis of *A*_1g_ mode. The intensity of samples is lower than that of the bare sapphire (0001) as shown in Fig. 6b. Initially, for the samples with lower initial film thickness from 2 to 20 nm, intensity is drastically reduced along with the formation of highly dense dome-shaped Ag NPs. When the initial film thickness is varied from 30 to 40 nm, the peak intensity is gradually increased where various configurations of irregular nanocluster are formed. Later, for the higher deposition thickness, intensity is consistently decreased with the formation of the nanocluster network. From this observation, it should be noted that the Raman intensity varies likely due to the change in surface coverage of various Ag nanostructures. At the same time, the peaks are slightly right-shifted as compared to the bare sapphire as shown in Fig. 6c, which is the consequence of the stress produced by the lattice mismatch between the Ag nanostructures and sapphire (0001) [40, 41]. And, the FWHM is almost similar with the bare sapphire (0001) except slightly right-shifted for the samples with 2 and 20 nm that indicates the peak shape modulation. The full range Raman spectra are presented in Fig. S6, and the specific values of peak intensity, peak shift, and FWHM are summarized in Table S3.

Figure 7 represents the reflectance spectra of samples with respect to the film thickness. The reflectance spectra were analyzed within the range of 250–1100 nm including UV, visible, and NIR regions. For the bare sapphire (0001), the average reflectance is measured as 8.97%. Depending on the evolution of Ag NPs, merged NPs, and nanoclusters network, the reflectance curve shows distinct shape and average reflectance. As shown in Fig. 7a, correspondent to the Ag NPs phase between 2 and 20 nm, the average reflectance is gradually increased from 7.64% to 33.92% as the dome-shaped NPs size is increased with respect to the film thickness. At the deposition of 2 nm, highly compact small Ag NPs can cause the maximum forward scattering of photon, which cannot be detected. As a result, average reflectance can be lower than that of bare sapphire (0001). For 6–20 nm samples, the development of peaks in the reflectance signals was observed within the spectral range from 400 to 700 nm, whereas reflectance minima were observed in longer wavelength. Meanwhile, the peaks are gradually red-shifted from ~507.53 to ~536.15 nm as the average size of Ag NPs is gradually increased. The high reflection exhibited by the samples within visible spectral range can be attributed to the backscattering of the Ag NPs, which increases with the increased NPs’ size. At the same time, much lower reflectance was observed in the NIR (~650–1000 nm) region, which can be explained by the substrate influence on the angular scattering from the NPs that can randomize the direction of reflected light [42, 43]. Moreover, the plasmon resonance effect of the Ag NPs can significantly reduce the reflectance within the NIR region [44]. With the growth of merged nanostructures, the reflectance is gradually decreased with the higher initial film thickness of Ag. As the density of Ag NPs is decreased and the spacing between the adjacent nanoclusters has been increased with the higher film thickness, less amount of backscattering can be expected as the surface coverage is reduced [45]. Furthermore, the shape transformation from the dome to the irregular can influence the backscattering and hence lower the reflectivity [42]. In the growth phase of Ag nanoclusters network evolution with the deposition thickness varied between 40 and 100 nm, the reflectance is again gradually increased. The increase in the average reflectivity can be attributed to the increased size of Ag nanoclusters. The specific average reflectance values with respect to the film thickness are summarized in Table S2.

**Fig. 8:**
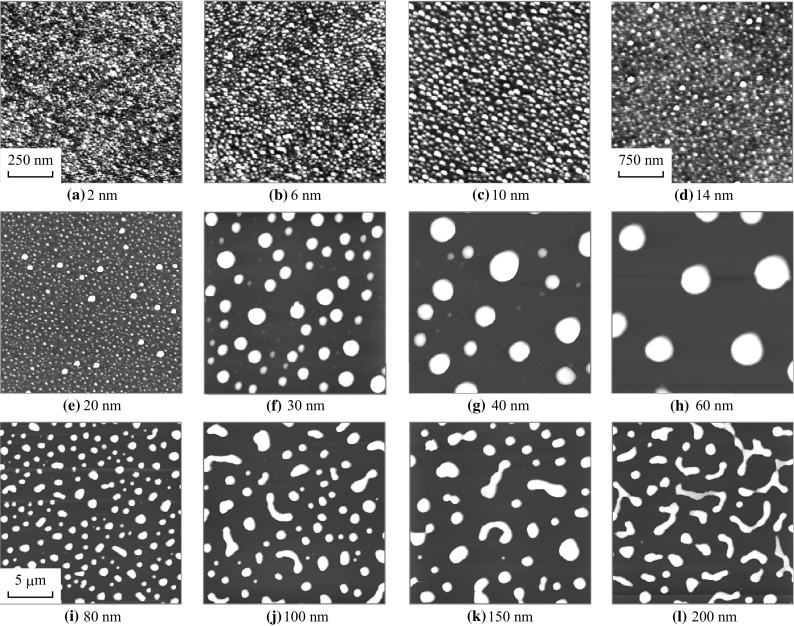
Evolution of tiny NPs, large round NPs, and coalesced NPs annealed at 750 °C for 180 s. AFM top-views of 1 × 1 µm^2^ in **a**–**c**, 3 × 3 µm^2^ in **d**–**h**, and 20 × 20 µm^2^ in **i**–**l**

**Fig. 9:**
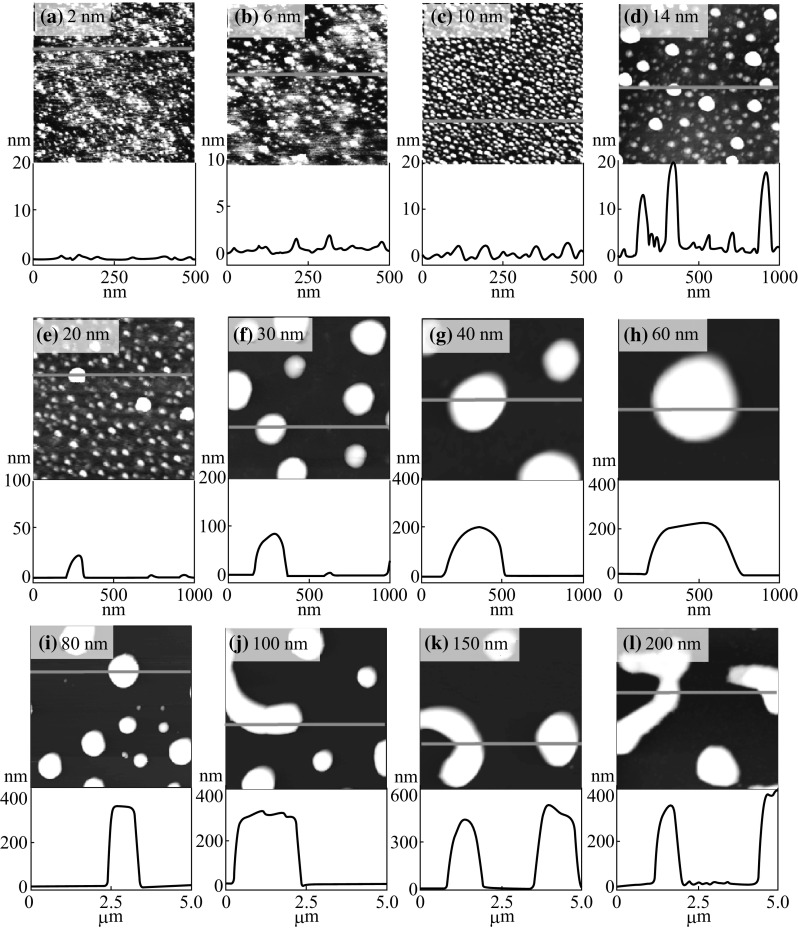
Morphological evolution of various-sized isolated Ag NPs to merged Ag NPs by the variation of deposition amount between 2 and 200 nm after annealing at 750 °C for 180 s. Corresponding line profiles show the height of the nanostructures. X-axes of the line profiles indicate the image size

**Fig. 10:**
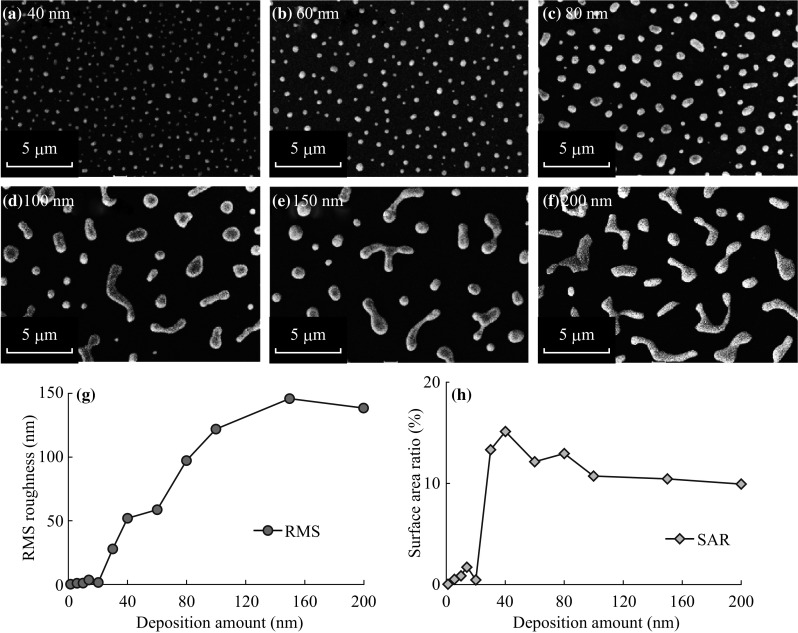
**a**–**f** SEM images of the Ag nanostructures on sapphire (0001) at 750 °C for 180 s. **g** RMS roughness (*R*_q_) and **h** surface area ratio (SAR) with respect to the deposition amount

Figures 8, 9, and 10 show the evolution of Ag nanostructures at higher annealing temperature (750 °C) for 180 s and identical range of initial film thickness from 2 to 200 nm. The morphological evolution of Ag nanostructures drastically differs from the lower annealing temperature (550 °C). As the surface diffusion of Ag thin film can be significantly enhanced [46] as well as the sublimation of Ag, the formation of distinct configuration of Ag nanostructures can be caused. The rate of sublimation directly depends on the temperature and equilibrium vapor pressure of the system as given by the relation,

5 $$R_{\text{s}} = \, \left( { 3. 5 1 3 { } \times { 1}0 2 2} \right) \, \left( {T \times M_{\text{Ag}} } \right)^{ - 1/ 2} \times P_{\text{eq}}$$

where the *T*, *M*_Ag_, and *P*_eq_ are the annealing temperature, molecular weight of Ag, and equilibrium vapor pressure [24]. From Eq. 5, it can be deduced that the rate of sublimation accelerates with increase in temperature because the term *P*_eq_ increased exponentially with temperature. Furthermore, the surface morphology of the Ag NPs also affects the sublimation process as expressed by the relationship with the radius of curvature (*r*) of Ag NPs

6 $$P_{\text{r}} = P_{\infty } { \exp }\left( { 2\gamma M_{\text{Ag}} /rT\rho R} \right)$$

where *P*_∞_, γ, ρ, and *R* are the equilibrium vapor pressure over a flat surface, isotropic surface energy, density, and gas constant, respectively [47]. From Eq. 6, it can be deduced that the radius of curvature of Ag nanostructures has the inverse relationship with the sublimation process which signifies the NPs with small (*r*) sublimate faster than NPs with large (*r*). As shown in Fig. 8a, the surface morphology with 2 nm deposition is almost similar to the bare sapphire except for the few tiny granular structures, which can be due to the significant sublimation of Ag at 750 °C.

At the 6 and 10 nm of initial film thickness, the surface developed with the formation of highly dense tiny NPs. For the further deposition variation between 14 and 20 nm, few relatively large Ag NPs are distinguished along with the tiny NPs on the background. With increased deposition between 30 and 80 nm, the fabrication of round dome-shaped Ag NPs is witnessed as shown in Fig. 8f–i. With the further increased initial film thickness to 100 and 200 nm, the Ag NPs merge and irregular Ag NPs are fabricated. More detailed analysis along with the enlarged views of AFM images and corresponding line profiles is presented in Fig. 9. At 2 nm of film thickness, the line profile shows almost flat surface profile, and for 6 nm few up and down are observed caused by the Ag NPs. The average height of cross-sectional line profiles is consistently increased with the initial film thickness, which represents the dimensional enhancement of surface morphology due to the formation of Ag NPs and merged nanostructures. The large-scale surface configuration at relatively higher Ag thickness can be witnessed on the SEM images in Fig. 10a–f. In addition, large-scale AFM top-views are presented in Figs. S7 and S8.

Surface enhancement can be equivalently discussed on the basis of surface parameters, such as RMS roughness (*R*_q_) and surface area ratio (SAR) as represented in Fig. 10g, h. As the surface consists of Ag NPs, whose dimensions are gradually increased with the added initial film thickness, the magnitude of *R*_q_ also consistently increased. However, the SAR value increases up to 40 nm due to the formation of round dome NPs with the enhanced dimension and gradually decreased for higher thickness from 30 to 100 nm. The *R*_q_ and SAR values are summarized in Table S1. The elemental characterization by EDS spectra as shown in Fig. S9, indicates the significantly low counts of Ag Lα1 due to the Ag sublimation at 750 °C. For a comparison with 200 nm sample, at 550 °C the Ag Lα1 count is ~14,494, whereas at 750 °C the count is ~2028. Similarly, for other corresponding samples at 750 °C, the Ag Lα1 counts are significantly lower than that of 550 °C due to sublimation.

**Fig. 11:**
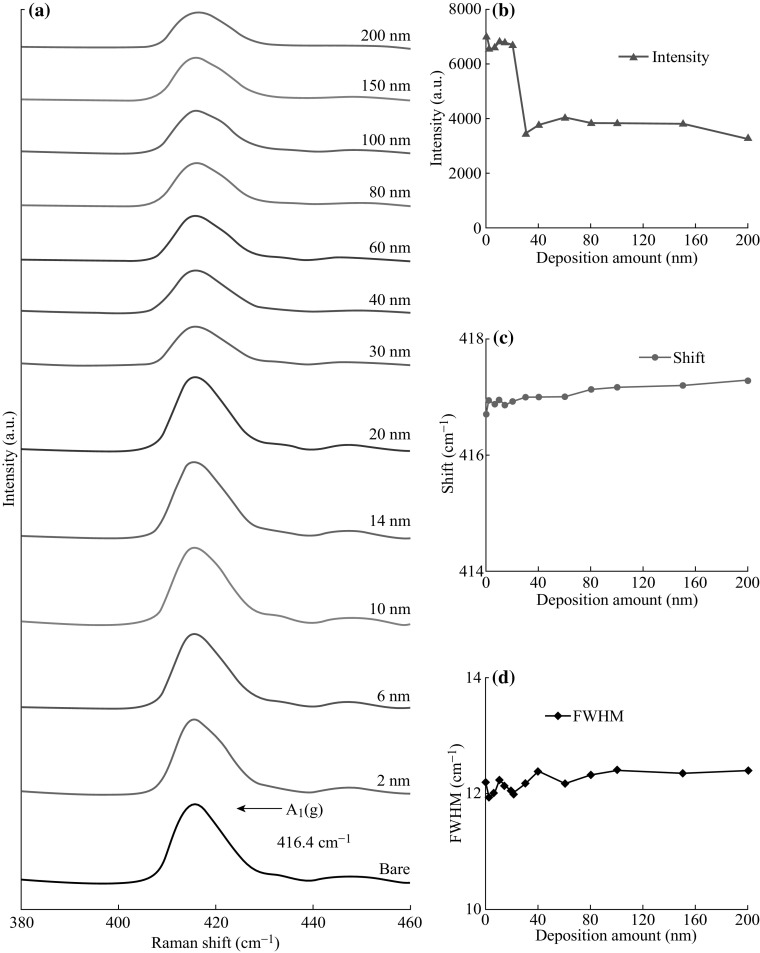
Raman spectra of the samples with various Ag nanostructures fabricated at 750 °C for 180 s. **a** Spectral range between 380 and 460 cm^−1^ and the *A*_1_(g) phonon mode at 416.4 cm^−1^. **b–d** Summary plot of peak intensity, peak shift, and FWHM with respect to the deposition amount

**Fig. 12:**
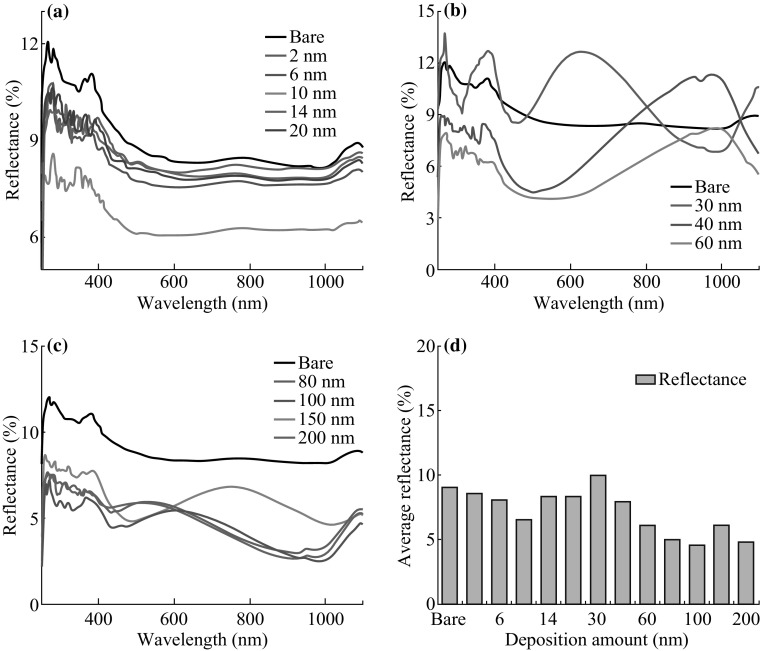
Reflectance of samples as a function of the Ag deposition amount annealed at 750 °C for 180 s. Reflectance spectra: **a** 2–20, **b** 30–60, and **c** 80–200 nm. **d** Average reflectance with respect to the deposition amount

From the optical characterization by the Raman spectra shown in Fig. 11, the intensity up to 20 nm is comparable to the bare sapphire as the Ag is sublimated at 750 °C of annealing and only tiny NPs remain on the substrate with negligible surface coverage. When the deposition thickness is increased from 30 to 200 nm, as clearly observed in the AMF top-views in Fig. 8f–l, the formation of widely spaced round and merged NPs is fabricated, which result in the higher surface coverage and hence the Raman intensity is significantly decreased. The full range Raman spectra are presented in Fig. S10. Moreover, from the reflectance spectra shown in Fig. 12, the reflectivity behavior is also significantly changed as compared to the 550 °C set. As shown in Fig. 12a, the reflectivity between 2 and 20 nm is recorded lower than the bare sapphire (0001), which can be due to the antireflective nature of the tiny NPs [48]. After the deposition of 30, 40, and 60 nm, with the formation of widely spaced round Ag NPs, the peaks in the reflectance spectra were observed and gradually red-shifted from shorter to longer wavelength along with the increased NP size as shown in Fig. 12b. When the initial film thickness increases from 80 to 200 nm, the reflectance is slightly reduced as shown in Fig. 12c. As discussed earlier, the overall change in the reflectance spectra and shift can be the consequence of different parameters such as shape, size, and configuration as well as the average surface coverage of Ag NPs.

## Conclusions

In summary, we demonstrate various configurations of Ag nanostructures on sapphire (0001) including NPs, irregular nanoclusters, and nanoclusters network formed at different annealing conditions and dependent on the variable thickness of Ag thin film. Based on the control of initial film thickness between 2 and 200 nm at 500 °C of annealing, three distinctive growth regimes of Ag nanostructures were observed: Specifically, the tiny to the enlarged dome-shaped Ag NPs between 2 and 20 nm, the merged and irregular nanoclusters between 30 and 60 nm, and the Ag nanoclusters network between 80 and 200 nm. The evolution of Ag nanostructures is symmetrically analyzed on the basis of surface diffusion, Volmer–Weber growth model, coalescence, and surface energy minimization mechanisms. The identical range of film deposition range was studied at higher annealing temperature (750 °C), which shows distinctive evolution of Ag nanostructures as a result of thickness-dependent characteristics of dewetting phenomena as well as the significant sublimation of Ag. Furthermore, the morphology dependence optical properties of Ag nanostructures were probed by the Raman and reflectance spectra analysis.

## Electronic supplementary material

Below is the link to the electronic supplementary material.

Supplementary material 1 (PDF 2352 kb)
