# Supplementary material for: Various Silver Nanostructures on Sapphire Using Plasmon Self-Assembly and Dewetting of Thin Films
Source: Nanomicro Lett. 2016 Nov 28;9:17. doi: 10.1007/s40820-016-0120-6 (PMC6225926; doi:10.1007/s40820-016-0120-6)
Supplement: Supplementary file 1 — Supplementary material 1 (PDF 2352 kb) [file 40820_2016_120_MOESM1_ESM.pdf]

Supplementary Information for

Various Silver Nanostructures on Sapphire Using Plasmon Self-Assembly and Dewetting of Thin Films

Sundar Kunwar[1], Mao Sui[1], Quanzhen Zhang[1], Puran Pandey[1], Ming-Yu Li[1], Jihoon Lee[1,2,]*

[1]College of Electronics and Information, Kwangwoon University, Nowon-gu Seoul 01897, South Korea

[2]Institute of Nanoscale Science and Engineering, University of Arkansas, Fayetteville AR 72701, USA

*Corresponding author. E-mail: jihoonleenano@gmail.com

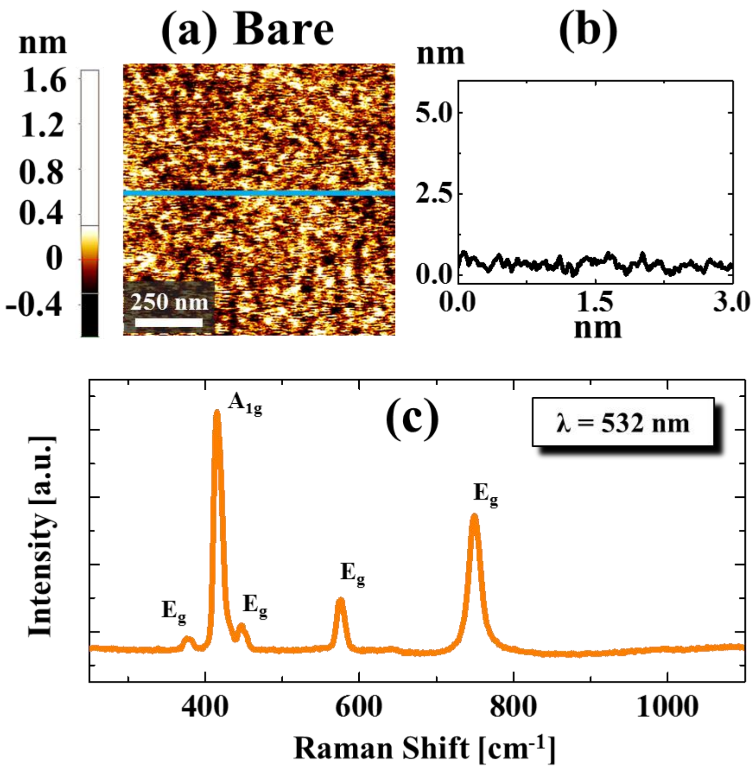

Fig. S1 a Surface morphology of bare sapphire (0001) after degassing. **b** Cross-sectional line profile in reference to the line in **a**. **c** Raman spectrum of bare sapphire depicting the five vibration modes as labeled at 416.53 (A_{1g}), 378.24, 446.83, 575.78, and 749.65 cm^{-1} (Eg). The spectra were measured under the excitation of 532 nm with 220 mW at an ambient condition

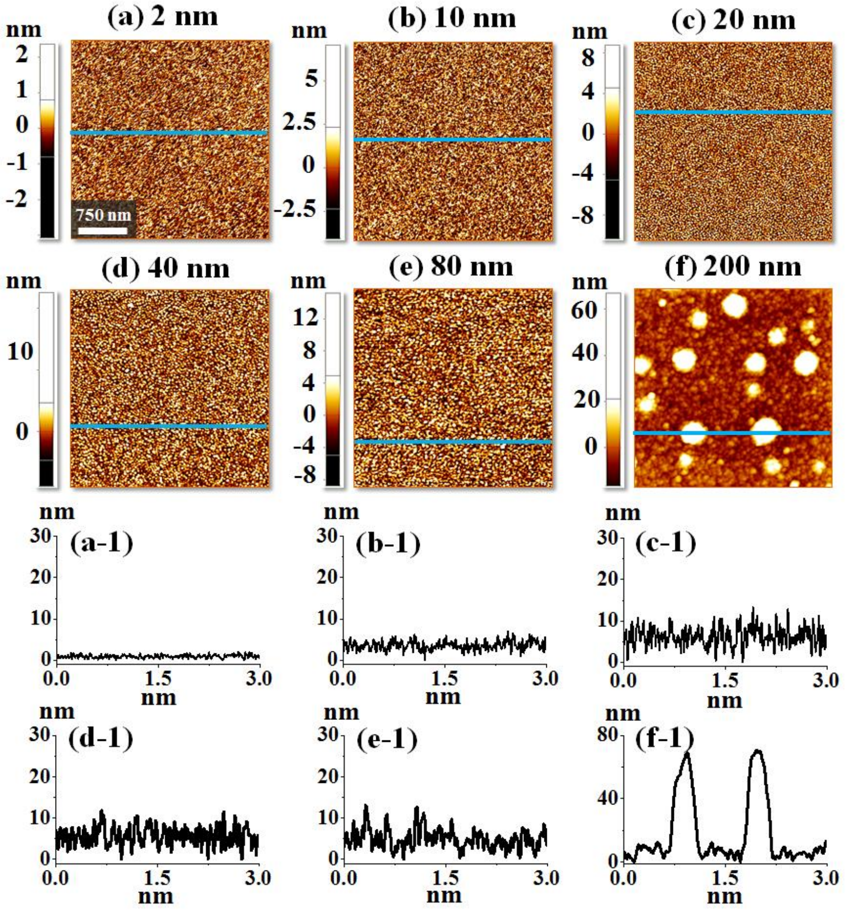

Fig. S2 a-f Surface morphology evolution of the samples before annealing, deposited with the various thickness of Ag between 2 and 200 nm as labelled. (**a-1**)-(**f-1**) Cross-sectional line profiles in references to the AFM top-views in **a-f**

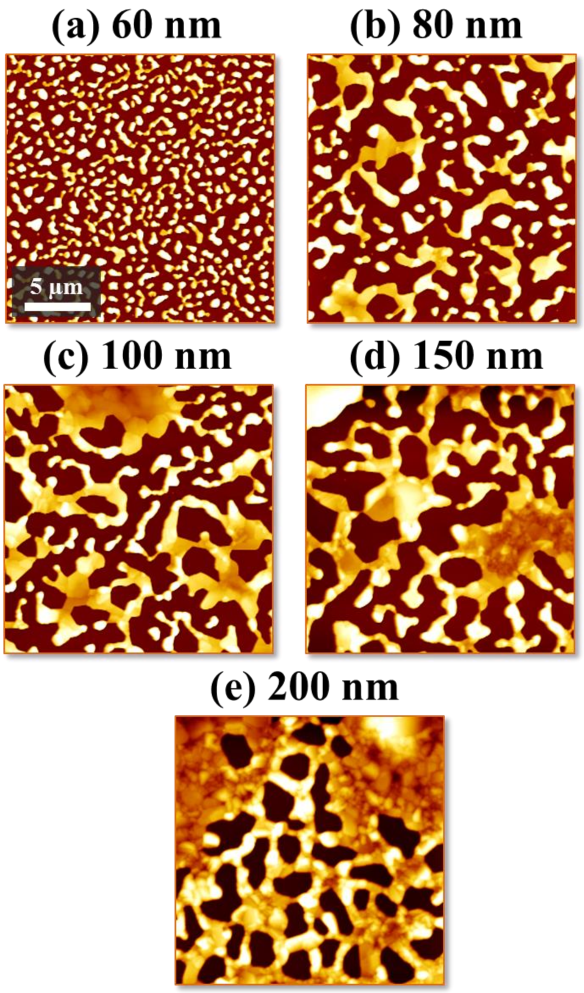

Fig. S3 Evolution of nanoclusters networks in large-scale AFM top-views: annealed at 550 °C for 180 s with the deposition amount variation between 60 and 200 nm

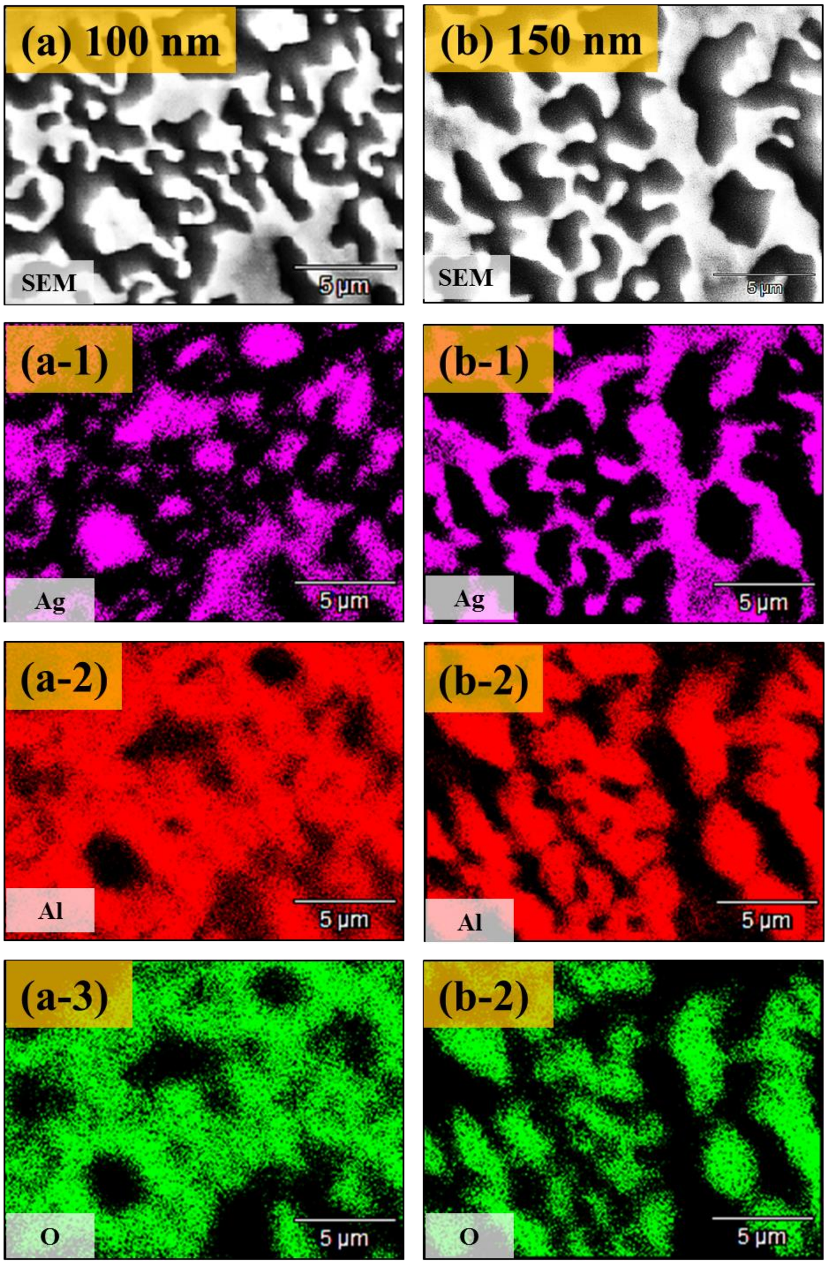

Fig. S4 EDS phase maps showing the distinct phase of elements (Ag, Al, and O) presented in the samples with the deposition of 100 nm (**a** - panel) and 150 nm (**b** - panel) annealed at 550 °C for 180 s

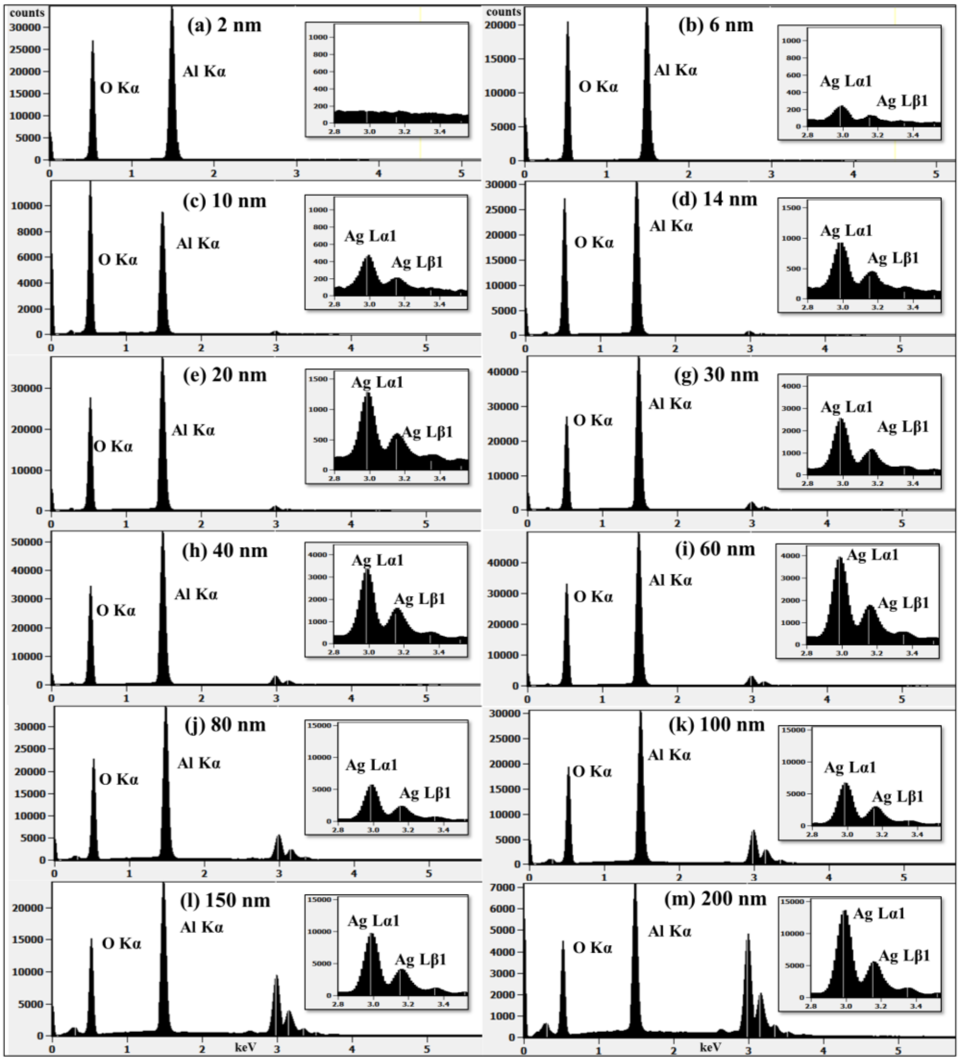

Fig. S5 EDS spectra of the samples within the range of 0-5.5 keV annealed at 550 °C for 180 s with the deposition amount variation from 2 to 200 nm. Insets show the detail of the Ag peaks between 2.8 – 3.4 keV

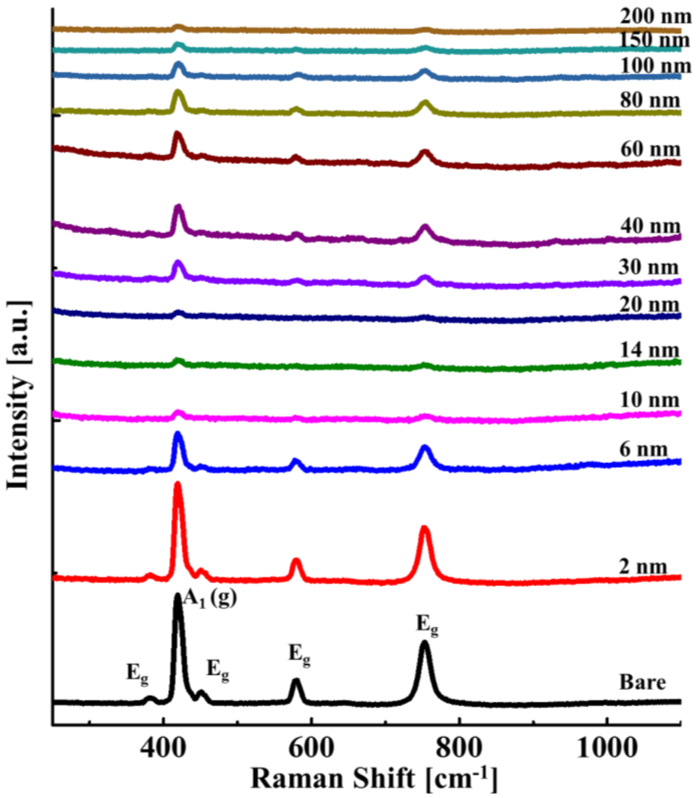

Fig. S6 Raman spectra of the samples annealed at 550 °C for 180 s by controlling the deposition amount as labelled (2-200 nm)

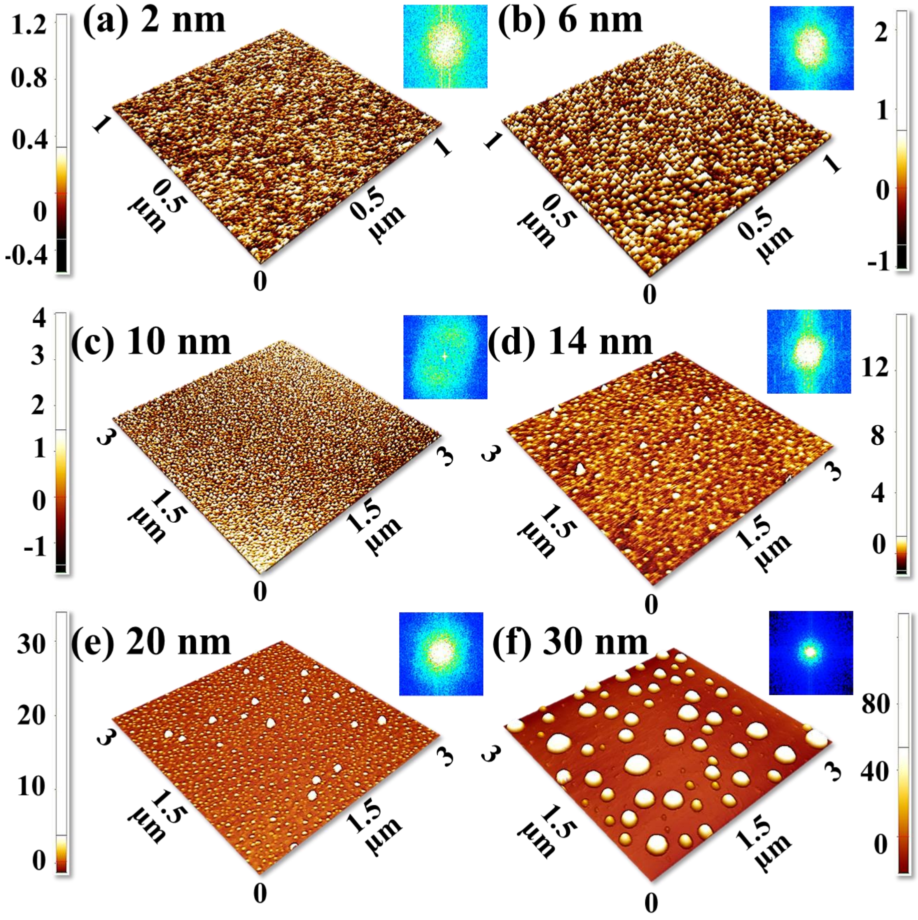

Fig. S7 AFM 3D-side views of tiny compact to large isolated Ag NPs on sapphire (0001) after annealing at 750 °C for 180 s by the variation of deposition thickness from 2 to 30 nm. Insets show the FFT power spectra of the corresponding AFM images

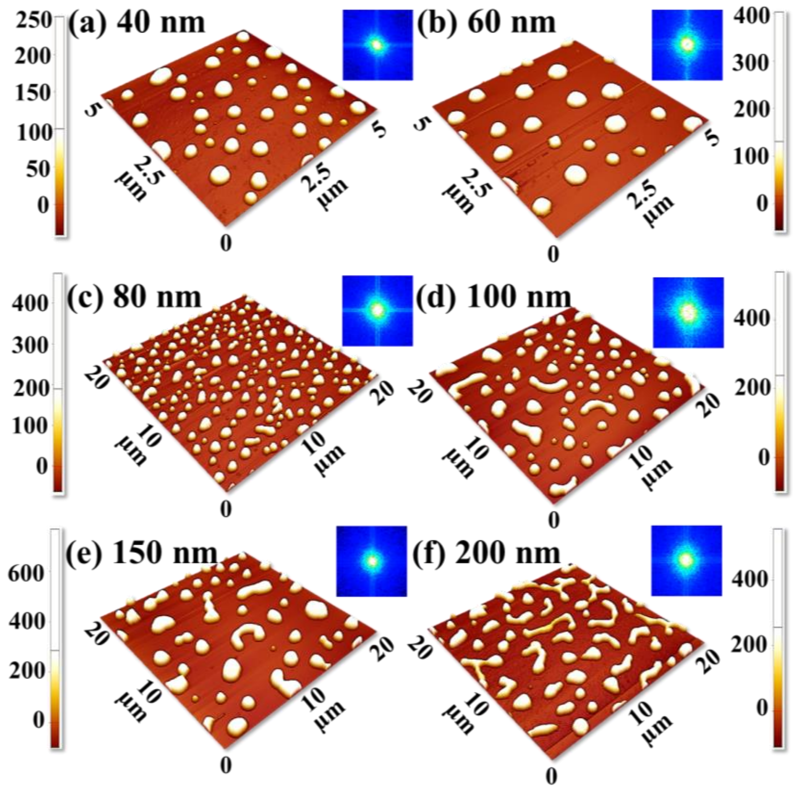

Fig. S8 Evolution from large isolated Ag NPs to the merged Ag nanostructures on sapphire (0001) by the control of deposition amount (40-200 nm) annealed at 750 °C for 180 s. Insets represent the 2D FFT power spectra of corresponding AFM images

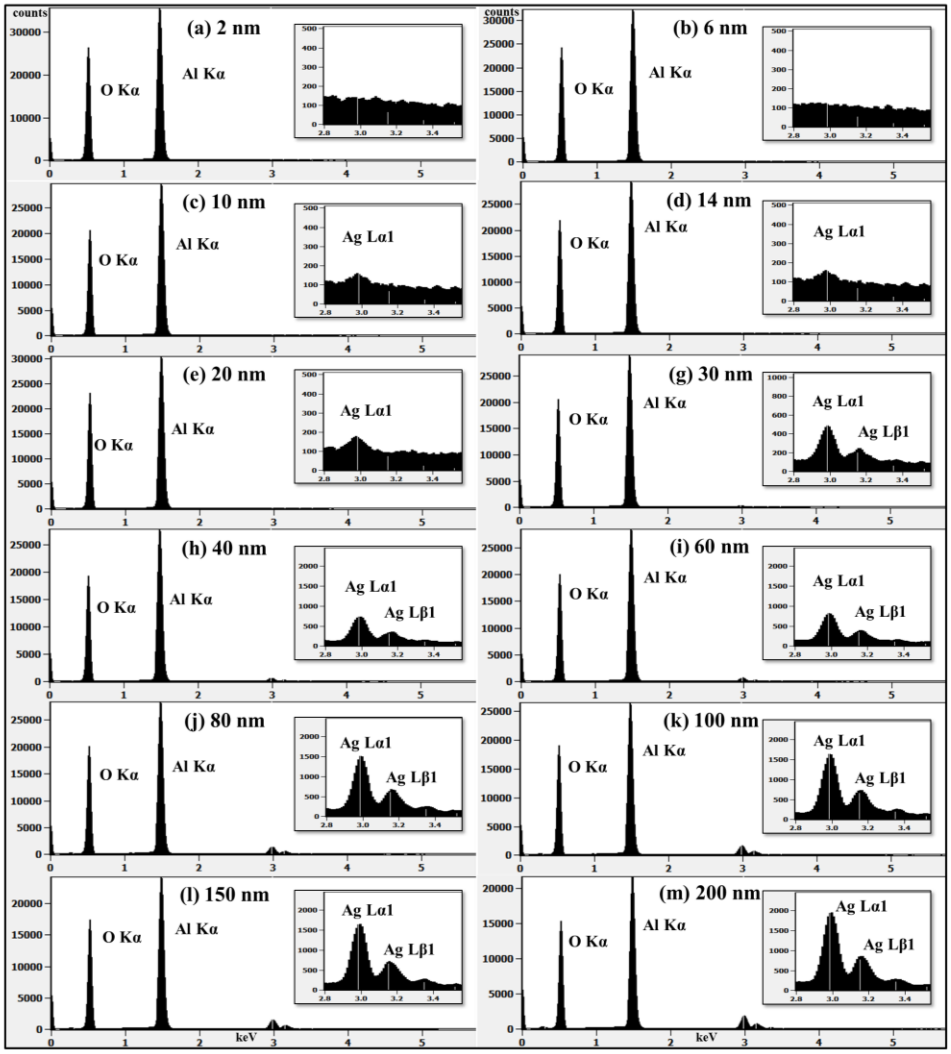

Fig. S9 Full range EDS spectra showing the Al, O, and Ag peaks within 0-5.5 keV of samples annealed at 750 °C for 180 s with the deposition amount as labelled. The enlarged views of Ag Lα1 and Ag Lβ1 are presented as insets

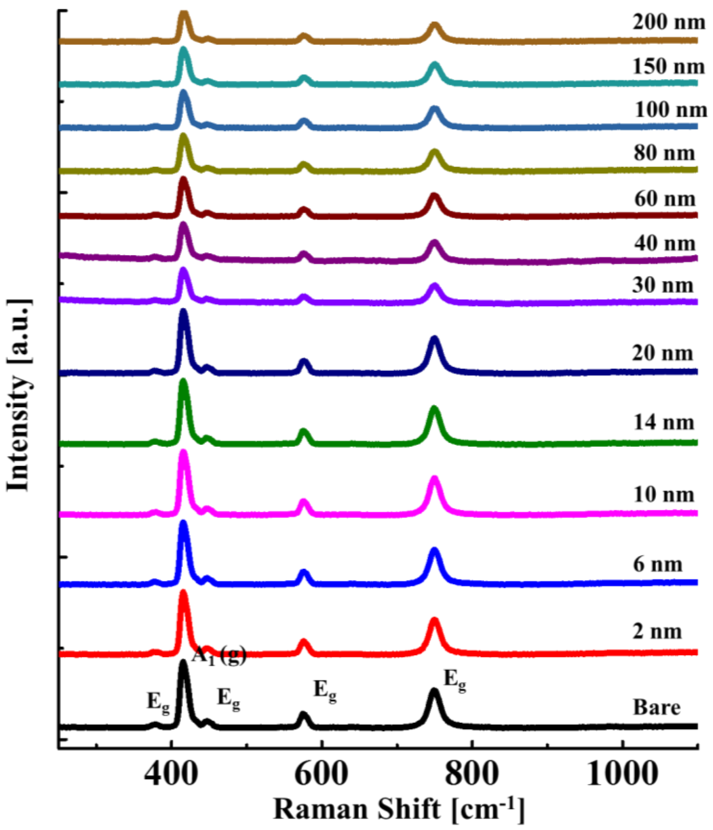

Fig. S10 Raman spectra of the samples fabricated with various Ag nanostructures at 750 °C for 180 s by the Ag thickness between 2 and 200 nm

Table S1 Summary of RMS roughness (*R*q) and surface area ratio (SAR) of the samples annealed at 550 and 750 °C for equal 180 s with the variation of deposition thickness between 2 and 200 nm

**Deposition Amount [nm]**	**Annealing Temperature (°C)**			
	**550 °C**		**750 °C**	
	**Rq (nm)**	**SAR (%)**	**Rq (nm)**	**SAR (%)**
**2**	1.522	5.18	0.19	0.13
**6**	2.049	8.53	0.48	0.54
**10**	4.63	20.18	0.74	0.85
**14**	9.34	20.17	3.45	1.72
**20**	13.52	24.60	1.38	0.49
**30**	36.88	23.1	27.46	13.33
**40**	55.51	19.82	51.78	15.15
**60**	67.86	14.70	58.69	12.14
**80**	94.82	11.76	96.90	12.97
**100**	92.87	10.1	121.75	10.75
**150**	127.67	11.78	145.75	10.46
**200**	121.1	9.34	138.47	9.93

Table S2 Summary of average reflectance with respect to the deposition amount from 2 to 200 nm after annealing at 550 and 750 °C for 180 s

Deposition Amount [nm]	Reflectance (%)	
	550 °C	750 °C
**Bare**	8.97	8.97
**2**	7.64	8.52
**6**	14.15	8.04
**10**	25.35	6.52
**14**	23.88	8.31
**20**	33.92	8.28
**30**	19.76	9.96
**40**	12.50	7.89
**60**	4.36	6.04
**80**	3.33	4.94
**100**	4.76	4.49
**150**	9.49	6.06
**200**	14.08	4.76

Table S3 Summary of Raman $A_1(g)$ phonon mode at 416.4 cm^{-1}: peak intensity, peak shift and FWHM of the samples with the variation of deposition amount from 2 to 200 nm, annealed at 550 and 750 °C for 180 s

**Deposition Amount (nm)**	**Annealing Temperature (°C)**					
	**550 °C**			**750 °C**		
	**Intensity (a.u.)**	**Shift (cm^{-1})**	**FWHM (cm^{-1})**	**Intensity (a.u.)**	**Shift (cm^{-1})**	**FWHM (cm^{-1})**
**Bare**	6923.86	416.55	12.16	6923.86	416.55	12.16
**2**	6190.54	416.65	11.97	6567.96	416.94	11.94
**6**	2382.80	416.68	11.96	6647.11	416.87	12.00
**10**	419.00	416.07	13.33	6802.33	416.95	12.21
**14**	334.83	416.58	11.62	6764.18	416.86	12.10
**20**	259.44	417.05	11.94	6654.53	416.93	12.01
**30**	1120.49	416.85	12.75	3421.70	417.00	12.17
**40**	1827.66	416.98	12.00	3764.08	417.01	12.39
**60**	1558.09	416.81	12.16	4016.90	417.01	12.16
**80**	1367.32	416.66	12.32	3824.96	417.14	12.31
**100**	874.42	416.92	12.14	3811.80	417.17	12.39
**150**	423.92	416.20	12.52	3797.68	417.20	12.34
**200**	249.86	416.87	11.82	3272.51	417.29	12.39
